# Accessibility and Digital Mental Health: Considerations for More Accessible and Equitable Mental Health Apps

**DOI:** 10.3389/fdgth.2021.742196

**Published:** 2021-09-29

**Authors:** John Bunyi, Kathryn E. Ringland, Stephen M. Schueller

**Affiliations:** ^1^Department of Psychological Science, University of California, Irvine, Irvine, CA, United States; ^2^Computational Media Department, University of California, Santa Cruz, Santa Cruz, CA, United States; ^3^Department of Informatics, University of California, Irvine, Irvine, CA, United States

**Keywords:** digital mental health, accessibility, mental health, disability, access, mHealth

## Abstract

Digital mental health is often touted as a solution to issues of access to mental health care. However, there has been little research done to understand the accessibility of digital mental health, especially for those with disabilities. In this piece, we define accessibility as it relates to mental health apps, describe the current state of accessibility in the digital world broadly and in mental health apps more specifically, outline why accessibility matters in mental health apps, and identify future steps to better incorporate accessibility into research and development of mental health apps.

## Introduction

The number of mental health apps continues to rise. Estimates suggest that over 10,000 mental health apps are now available to the public (1). The types of mental health apps vary widely; some provide self-help resources grounded in evidence-based practices such as Cognitive Behavioral Therapy (CBT), others provide pathways to virtual care either from professional providers or lay peers, whereas still others are designed to augment therapy and increase the efficiency or efficacy of clinical care. As a subset of the broader digital mental health movement, apps are often seen as a bridge to accessing care, providing an easy and cost-effective way for consumers to get help through devices they may already use (2, 3). In short, to make mental health care more “accessible”.

However, this potential to expand access to mental health care to those in need is predicated on whether such products are truly accessible for diverse, underserved, and vulnerable populations. Many believe that accessibility means “making products usable by people in a wide range of situations— circumstances, environments, and conditions” (4), and that accessible design ultimately benefits everyone. While accessibility in digital mental health has often been considered in terms of expanding access to various populations including ethnic, racial, sexual and gender minorities (3, 5, 6), individuals across the lifespan (7), individuals experiencing homelessness, or rural populations (3), it has rarely been considered in terms of people with physical, sensory, and cognitive disabilities (8, 9). This is a missed opportunity in digital mental health, where researchers and developers could be leading the way in incorporating understanding of affective, behavioral, and cognitive aspects to create mental health technologies that might truly bridge the gap between those who are able to access and receive services and those who cannot.

In this paper we will a) define accessibility, especially as it pertains to mental health apps, b) present existing work around models of accessibility as it relates to technology and digital health, c) identify considerations specific to mental health apps, and d) recommend future directions in digital mental health to better incorporate accessibility. While digital mental health is a broad field which includes many cutting-edge technologies (e.g., virtual reality, wearables), we focus this paper on mental health software which run on commonly available platforms (e.g., personal computers, tablets, and smartphones). By acknowledging all facets of accessibility in design and use, digital mental health can better meet its promise to expand access and benefit broader audiences, including those who are most vulnerable and those most in need of mental health care.

To better understand the lens of the authors of this work, we disclose that some of the authors have personal experience with disabilities including navigating attention-related challenges as a person with Attention Deficit/Hyperactivity Disorder. Much of our interest in writing this piece came from our experience with reviewing mental health apps and noticing a lack of apps that respond to system-level accessibility tools (e.g., captions, screen readers, etc.), our work in evaluating the implementation of digital mental health in various service settings, and research studying and designing assistive technologies for people with disabilities. We also consulted colleagues who are mental health providers with mobility/dexterity and visual impairments.

## Defining Accessibility

For a resource to be most accessible, it must be able to be used by a person with a disability for the same purpose, the same effectiveness, and with a similar amount of time and effort as someone who is non-disabled (10). In the context of the physical world, this can take the form of ramps or braille signs. In the context of the digital world, accessibility means that a website or tool is built with content and design that is understandable and navigable with or without assistive technologies. It also means that a digital resource includes back-end technical or coding considerations, such as compatibility with assistive technologies to allow for greater access for those with disabilities.

The Web Accessibility Initiative (WAI) provides an example of guidelines to define what constitutes an accessible digital resource. The POUR guidelines highlight considerations such that people with disabilities can perceive, understand, navigate, interact with, and contribute to the web (11). Considerations within POUR include: ***P***erceivable information and user interface; ***O***perable user interface and navigation; ***U***nderstandable information and user interface; and ***R***obust content and reliable interpretation (12).

**Figure 1:**
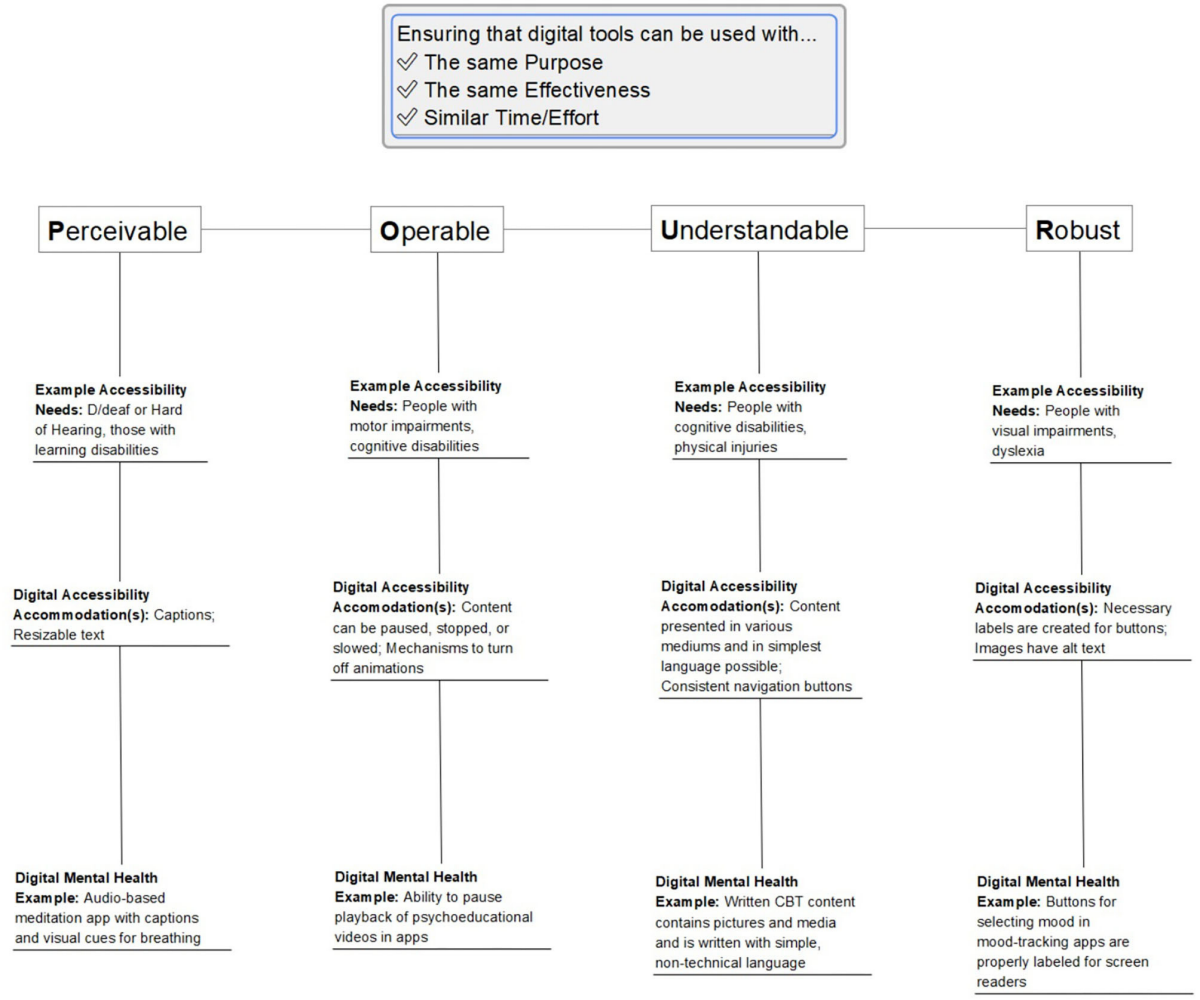
Applying the Web Accessibility Initiative (WAI) POUR guidelines to digital mental health.

One challenge with defining accessibility with regards to apps, and with developing accessible mental health apps, is that accessibility needs vary dependent on a person's disabilities. People, for example, who are D/deaf or hard of hearing, or those with learning disabilities may benefit from captioning. People who have vision impairments or those with Attention Deficit/Hyperactivity Disorder (ADHD) may benefit from text customization options such as text size, scaling, contrast, and reflow. People with color blindness may benefit from images that use additional formatting or annotations to help display information. Others who have motor impairments or people with cognitive disabilities may benefit from tools such as voice dictation and eye-tracking to help interface with technology. More examples are provided in Figure 1.

Accessibility for mental health apps requires making similar considerations as one would in the broader digital world. For example, a meditation app designed with accessibility for diverse needs in mind would include captions for audio-based meditations and audio indicators for visual cues such as breathing. The importance of such considerations is magnified for a population that is already dealing with layers of challenges to accessing the care they need. To better understand accessibility as it relates to digital mental health, we can start by looking at existing work in digital accessibility and in digital health.

## Existing Work Focusing on Digital Accessibility and Digital Health

In general, accessibility within the digital space has taken steps toward standardization through organizations and protocols such as WAI and its related efforts (13, 14). Researchers, organizations, and major tech companies such as Microsoft and Mozilla, have contributed to the creation of accessibility standards and the creation of guidelines for assessing the accessibility of websites and apps such as WAI's POUR guidelines (12, 15–18). Mobile platforms such as iOS and Android are also designing with physical and sensory accessibility in mind. To encourage app developers to code their apps to work with assistive technologies, Apple (iOS) and Google (Android) have been actively integrating assistive technology such as screen readers into their operating systems (19, 20). Apple, Google, and several other organizations have also created automated tools that can assess an app and check if it has integrated accessibility features or met certain guidelines (21). This helps to standardize and ease the burden of checking for accessibility in technology (although human review is still warranted). Despite all these efforts, accessibility in the digital world is still poor, including in digital health (22). However, as researchers and designers in this space, we should be leading the ways in terms of accessibility because everyone experiences mental health challenges at some point in their lives – ranging from acute to serious mental health discomfort or distress. As such, making more mental health resources available to everyone should be a common goal.

Research on accessibility remains limited in the digital mental health space. Work on accessibility in digital health more broadly is more developed (23–26) and offers some insights on how to better consider and increase accessibility in mental health apps. A number of studies in digital health have found that principles of co-design or including those with lived experience in developing an app can help identify and address accessibility issues for an app's target audience such as customization and personalization, inclusive language, or better measurements based on differing abilities (27–33).

One study looking at the use of digital health apps for vision impaired individuals found that by applying accessibility focused user experience (UX) guidelines to an app's design, information recognition and uptake increased for both those who are vision impaired, and those who are without vision impairment (27). The study's researchers also found both low-quality information presented in an app, as well as poor presentation and organization of valid information across other apps. Researchers point out that this can be particularly troubling especially for low-vision consumers, for whom information may already be difficult to access.

One approach in research is to include feedback from caregivers or professionals, *in addition* to feedback from disabled consumers (28). Done appropriately, which means including caregiver and professionals to provide additional, diverse viewpoints and triangulation with consumer voices, this can be useful to add context and insights (29, 34). However, professionals may have worked with other people with similar disabilities but differing accessibility needs. Thus, although work in digital health has demonstrated potential usefulness of triangulation with caregivers and professionals, it needs to be done in a way that does not diminish the involvement and voice of disabled people.

Unfortunately, accessibility research in digital mental health itself is limited. Again, there is an opportunity here to understand and address accessibility barriers for those who might use mental health apps. In the following section we look at accessibility considerations for mental health apps as they relate to a disabled person's ability to use mental health apps for the same purpose, effectiveness, and with a similar amount of time and effort as someone who is without disability.

## Accessibility Considerations in Mental Health Apps

A mental health app may not fulfill its intended purpose without weighing accessibility in design. Mental health apps are a solution for receiving treatment for those unable to access traditional forms of therapy or an opportunity to enhance treatment for those who are already receiving therapy. Accessibility considerations for mental health apps should help ensure that a mental health app can perform the same functions, achieve the same outcomes, and require the same amount of time and effort for those with or without disabilities. This includes consideration of physical, sensory, and cognitive disabilities, as well as the intersection of such disabilities with mental health concerns (35).

Mental health apps should aim to perform the same function regardless of the accessibility needs of those using them. Given that major functions of mental health apps are often around providing psychoeducation through didactic material, reinforcing skills through interactive exercises, and supporting tracking of things such as mood, symptoms, triggers, or medication, apps should attempt to ensure that functions can be performed by all. Various issues, however, may interfere with consumers using these functions. For example, those who are D/deaf or hard of hearing may greatly benefit from captioning in audio-heavy meditation apps. Alternatively, those who are vision impaired may require resizable or customizable text when navigating text-heavy content. It is estimated that almost 33% of adults with physical disabilities in the U.S. (about 17.4 million people) experience mental health issues, and those with disabilities should be afforded the same benefits from digital tools as those who are non-disabled (36).

Those with temporary disabilities must also be able to perform the appropriate tasks within a mental health app. Those experiencing common side effects of psychotropic medications such as blurry vision, tremors, or memory impairments, may have trouble reading text-heavy content that is not resizable or customizable; an accessibility feature commonly needed by those who are vision impaired. In another example, over-animated or dense app designs may make it challenging to complete and retain didactic material for those with poor working memory, a symptom found within a variety of mental health challenges (37). Physical, sensory, and cognitive disabilities often co-occur with mental health conditions (38–40), adding layers of digital accessibility concerns for individuals. Creating accessible content should serve to benefit a mental health app's reach, while maintaining or even improving the app's effectiveness at delivering information.

For a mental health app to be of the same benefit to people with disabilities, they must be able to use the app for the same or similar amount of time and effort. In addition to the examples outlined above, poor adherence to accessibility guidelines can also impact the amount of time and effort required to use and trust a mental health app (41, 42). Failing to apply accessibility guidelines to privacy policies may result in overwhelmingly complex policies written at college-level reading levels requiring additional time and effort to understand (29, 43, 44). This is especially critical for those using a mental health app, who might need clear assurances that sensitive personal data such as what is shared in a mental health app will be treated with respect.

## Future Directions: Making Mental Health Apps More Accessible

Work on accessibility on mental health apps is sparse. Prioritizing such work, however, could provide an opportunity to expand the reach of mental health apps, especially to those who face many barriers to traditional mental health care. We note three key areas where we should work to improve the consideration of accessibility for mental health apps – standards, research, and recognition.

Various standards and evaluation frameworks have evolved for mental health apps [e.g., APA Framework (45), Enlight (46), One Mind PsyberGuide (47)] which have consensus around key areas of evaluation including evidence-base, user experience, and data security and privacy. However, none of these standards and frameworks consider accessibility. The closest aspect would be user experience, but although accessibility impacts user experience, they are not equivalent and assessing user experience may not identify accessibility issues. Accessibility should be a core area of evaluation for mental health apps. This could include accessibility as discussed in this paper, as well as other components contributing to access, such as language or system requirements, but its inclusion in evaluation would go a long way to help promote inclusion. It is beyond the scope of this paper to define specific, measurable items that define whether a mental health app is accessible or not, however, the examples provided in Figure 1 outline some considerations that might be incorporated in such items. A recent synthesis of various evaluation frameworks identified 11 distinct questions related to accessibility covering areas of availability, offline modes, and vulnerable populations as the target audience (48). Another review has recommended the Matching Person to Technology (MPT) model as one framework to use when considering health apps for people with intellectual disabilities (30).

Accessibility research needs to be collaborative, by including those with lived experience and accessibility needs in the design and iteration of mental health apps. Although as discussed earlier, providers and caregivers can provide additional data, this should be done in a way that empowers rather than diminishes the voices of those with lived experience. Iterative research is key because the diverse accessibility needs of consumers are unlikely to be addressed or incorporated in just a few focus groups or studies. Furthermore, accessibility considerations made during various stages of iterative research should be well described and contextualized to allow other research groups to iteratively build off each other's findings. Collaboration is also required between diverse stakeholders including industry partners to make use of advances in accessible technologies more broadly in research designs and considerations.

Qualitative research and interviewing geared toward understanding accessibility in mental health apps can begin to inform specific needs in the space. Similar work has been done by Bernard and colleagues, but that was focused more on how mental illness can affect accessibility of apps and websites (29). Research design that is mindful of participants' conditions is also important. Beaton and colleagues, for example, demonstrated consideration of how the temporary disabilities caused by concussion symptoms, might impact their findings (28). Rather than limiting findings to information collected during the interview, they encouraged participants to follow up with any additional thoughts after the interview to provide time for recall and processing.

Consumer surveys, especially those done early in the process of research or design, can provide valuable understanding of potential areas to explore including needs and opportunities. However, when using surveys, recruitment needs to ensure proper representation to consider accessibility. If survey samples are small or targeted toward specific populations, that population should be well-contextualized to ensure that findings are interpreted appropriately. If survey samples are large, and intended for wide-spread generalization, an eye toward proper representation of diverse accessibility needs should be a consideration in recruitment.

Elevating accessibility to a critical consideration in the digital mental health space requires making sure it is not brushed aside by simply stating that it is already included in current evaluations. Furthermore, professional organizations and journals could do a better job of raising accessibility issues, first by making these spaces more accessible, and second by ensuring that accessibility issues are included in the dialogue.

Furthermore, while this paper focused on mental health apps, it is equally important to consider accessibility in the broader digital mental health space, as many digital mental health tools are being made available across various device form factors. Finally, while we centered around accessibility in the context of disability, it would be wrong to ignore the importance of *usability* and *inclusion*. Usability refers to considerations for efficient and satisfying design, while inclusivity includes considerations for elements such as culture, education, and digital literacy in the development of technologies (49). Both are also important to consider in digital mental health, and they often overlap with each other and with accessibility. Without being mindful of “usable” design, we risk neglecting accessible design, and without creating “inclusive” content, we are limiting others' ability to access the content.

## Conclusion

Technology has facilitated the creation of a multitude of mental health apps. Over the past few years, accessibility has been a growing consideration in technology generally. The time is overdue for these areas to come together and promote accessibility within mental health apps. A first step to promoting accessibility would be the adoption of standards in mental health apps which follow principles from established accessibility guidelines. Second, research should explore whether mental health apps are usable for the same purpose, with the same effectiveness, and with the same time, across people. Third, the field needs to recognize that accessibility for some-but-not-all is counter to the goal of digital mental health to make resources broadly available and will only serve to entrench rather than overcome inequalities in care. Keeping the status quo and failing to prioritize the accessibility needs of consumers of mental health apps limits the quantity and quality of available treatment options. Furthermore, prioritizing accessibility may not only benefit individuals with disabilities but create better mental health apps for all users. With careful consideration and implementation of accessibility work within the field of digital mental health, we can make even bigger strides to deliver on the goals of digital mental health to increase access for all.

## Data Availability Statement

The original contributions presented in the study are included in the article/supplementary material, further inquiries can be directed to the corresponding author/s.

## Author Contributions

JB wrote the first draft of the manuscript. JB, KER, and SMS wrote sections of the manuscript. All authors contributed to manuscript revision, read, and approved the submitted version.

## Funding

SMS received funding from One Mind for the operation and management of One Mind PsyberGuide. The funder was not involved in the study design, collection, analysis, interpretation of data, the writing of this article or the decision to submit it for publication.

## Conflict of Interest

SMS has received consulting payments from Otsuka Pharmaceuticals for work unrelated to this manuscript and is on the Scientific Advisory Board for Headspace, for which he receives compensation. The remaining authors declare that the research was conducted in the absence of any commercial or financial relationships that could be construed as a potential conflict of interest.

## Publisher's Note

All claims expressed in this article are solely those of the authors and do not necessarily represent those of their affiliated organizations, or those of the publisher, the editors and the reviewers. Any product that may be evaluated in this article, or claim that may be made by its manufacturer, is not guaranteed or endorsed by the publisher.
